# Two Cases of Prurigo Nodularis in Hematologic Malignancies: Hodgkin Lymphoma and Waldenström Macroglobulinemia

**DOI:** 10.7759/cureus.78540

**Published:** 2025-02-05

**Authors:** Filipa Reis, Francisca Sarmento, Sara Sarmento, Marta Freixa

**Affiliations:** 1 Internal Medicine, Unidade Local de Saúde de Santa Maria - Hospital Pulido Valente, Lisbon, PRT

**Keywords:** hodgkin lymphoma, malignancy, paraneoplastic syndromes, prurigo nodularis, waldenström macroglobulinemia

## Abstract

Prurigo nodularis (PN) is a chronic inflammatory skin disorder marked by intense itching and firm, pruritic nodules, often linked to dermatologic, autoimmune, or systemic conditions, including malignancies such as hematologic cancers. We present two cases illustrating PN as a potential cutaneous manifestation of hematologic malignancies: a 48-year-old male with a history of psoriasis and persistent pruritus misdiagnosed as scabies, later found to have Hodgkin lymphoma, and an 86-year-old female with fatigue, anemia, and erythematous lesions diagnosed with Waldenström macroglobulinemia. These cases underscore the importance of recognizing PN as a symptom of underlying malignancy, enabling earlier diagnosis and treatment to improve outcomes.

## Introduction

Prurigo nodularis (PN) is defined by intense pruritus lasting for more than six weeks, a history of repeated scratching, and subsequent development of pruritic, elevated, firm, and nodular skin lesions, in a vicious itch-scratch cycle [1]. PN is a relatively rare condition, with an estimated prevalence of 72 per 100,000 in an epidemiologic study of US adults aged 18 to 64 years. There is some evidence that PN is slightly more common in females. The number of nodules in PN can range from several to over a hundred, often grouped and symmetrically distributed on the extensor surfaces of the extremities and trunk [2].

PN is more commonly seen in dermatological diseases such as atopic dermatitis (AD), psoriasis, and urticaria. A retrospective database analysis in England reported that among patients diagnosed with PN, 52.2% had comorbid AD. Nevertheless, it can be secondary to various systemic diseases. Its association with malignancies, such as Hodgkin or non-Hodgkin lymphoma and multiple myeloma, has been increasingly recognized in recent years. These diseases can manifest as PN, either as a primary cutaneous finding or as part of a systemic picture [3].

The pathophysiology of PN remains incompletely understood. Although AD and PN are both type 2 inflammatory diseases, recent transcriptomic studies have revealed that PN is separated from AD. PN does not harbor the strong type 2 response pattern that is typically found in AD but is rather characterized by stromal remodeling and neurovascular dysregulation [4-6]. Pruritus in PN is driven by neurogenic inflammation, where activation of sensory neurons releases pro-inflammatory cytokines and neuropeptides, such as substance P and calcitonin gene-related peptide, worsening pruritus and skin damage. Inflammatory mediators such as interleukins (ILs) and tumor necrosis factor (TNF)-α also play a central role in the chronic nature of the disease. In the context of lymphoma, these inflammatory pathways may be augmented by the systemic effects of the malignancy, leading to an increased burden of skin lesions and pruritus in affected individuals [7].

## Case presentation

Clinical case 1

**Figure 1:**
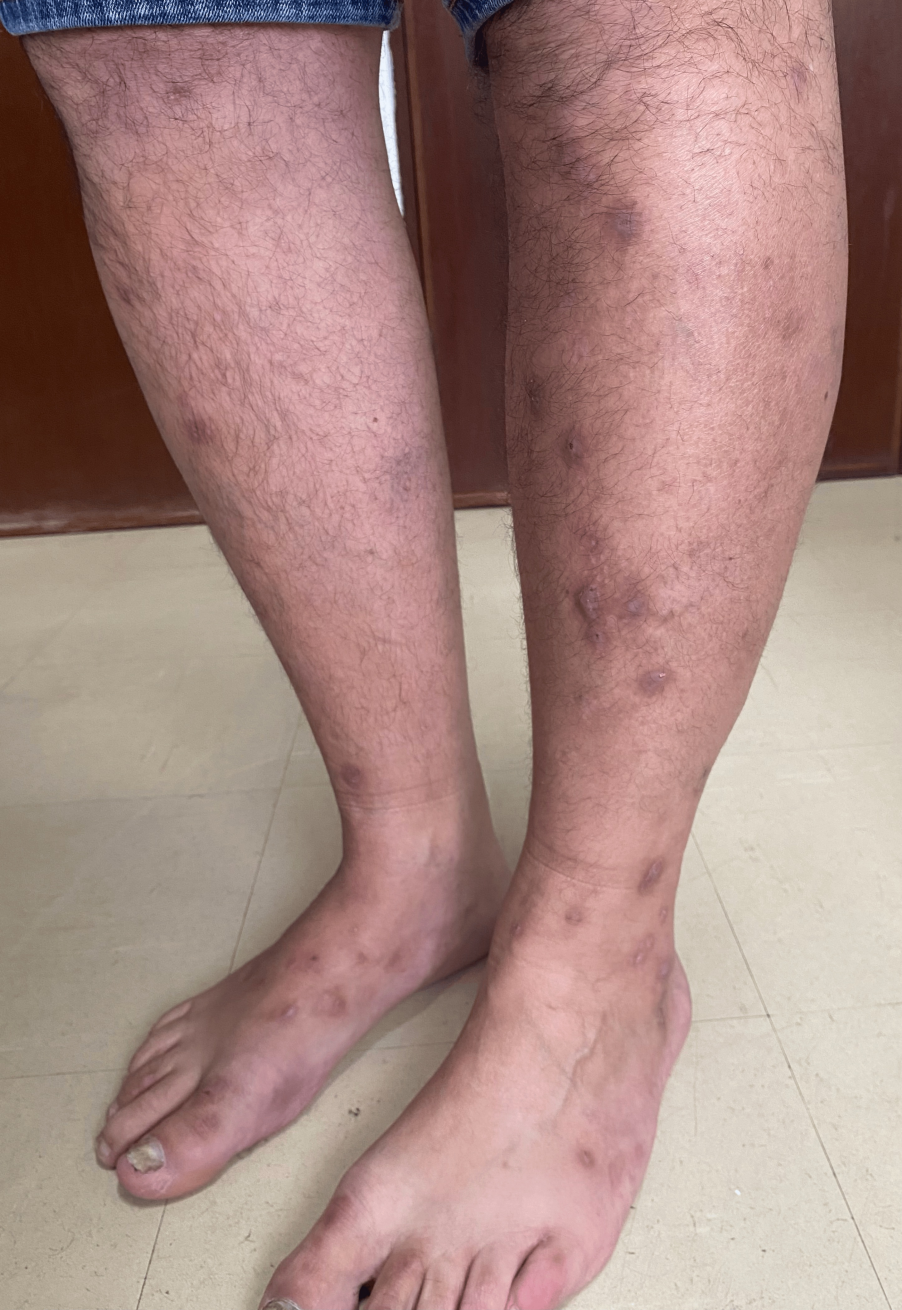
Lesions of prurigo nodularis in a patient with Hodgkin lymphoma.

A 48-year-old male, with a personal and family history of psoriasis, routinely medicated with topical clobetasol propionate. The patient was sent to an internal medicine appointment due to a clinical presentation characterized by a nine-month duration of severe pruritus and nodular skin lesions (Figure 1), weight loss (130 to 111 kg), and tiredness. He denied fever, night sweats, epigastralgia, anorexia, nausea, and acolia.

Skin lesions first appeared on the upper and lower limbs, mainly on the forearms, and later developed on the trunk. Lesions were accompanied by pruritus refractory to antihistamines and low-dose corticosteroids. The patient was initially treated under the presumptive diagnosis of scabies, with the administration of albendazole; however, the treatment proved ineffective. Improvement of pruritus was only achieved with prednisolone 60 mg/day and relapse occurred whenever the dose was reduced.

On physical examination, he presented several brownish papules all over the upper body and limbs, besides lesions compatible with scratching. A tense, non-painful mass, measuring approximately 2 cm in diameter, was identified in the right inguinal region, exhibiting limited mobility in relation to adjacent tissue planes.

Blood tests revealed an elevation in C-reactive protein (CRP) (2.7 mg/dL, normal range reference <0.5 mg/dL), while the white cell count remained within normal limits. Serological tests for HIV, hepatitis B, and hepatitis C were negative. Protein electrophoresis demonstrated no abnormalities. Thyroid and renal function tests were within normal ranges. Additionally, antimitochondrial antibodies were negative. Skin biopsy revealed circumscribed lichenification and ulceration, compatible with the clinical diagnosis of PN.

A CT scan revealed multiple lymphadenopathies located both above and below the diaphragm, as well as splenomegaly. Prompt excisional biopsy of the inguinal lymphadenopathy was performed, with histopathological analysis confirming the diagnosis of Hodgkin lymphoma, nodular sclerosis subtype.

The patient was followed up on an outpatient basis and referred to a hematology appointment, as the suspicion of lymphoma was very high. While awaiting the biopsy results, additional studies were conducted before starting chemotherapy.

Positron emission tomography imaging demonstrated increased radiotracer uptake in multiple lymph node regions, including an axillary nodule (standardized uptake value (SUV) 6), mediastinal and infracarinal nodes (SUV 10), paraesophageal and bilateral para-aortic nodes (SUV 12-14), retrocrural nodes (SUV 15), paravertebral nodes (SUV 12), abdominal nodes (SUV 10), ileopelvic nodes (SUV 24), obturator nodes (SUV 23), as well as inguinal adenopathies (SUV 13). Additionally, a hypermetabolic mass was identified within the iliopsoas muscle (SUV 7). These findings were consistent with an advanced-stage disease, with an International Prognostic Score of 2.

Treatment was initiated with the ABVD chemotherapy regimen, consisting of doxorubicin, bleomycin, vinblastine, and dacarbazine. Following the first cycle of chemotherapy, there was a complete resolution of prurigo. The associated skin lesions progressively regressed and fully disappeared over the course of three months.

Clinical case 2

An 86-year-old female, with no previously known diseases and no routine medication, was referred to an internal medicine consultation due to complaints of fatigue, weakness, and erythematous nodular skin lesions that had developed over the previous three months. The patient also reported occasional headaches but denied weight loss, fever, night sweats, or other symptoms. Additionally, she had not taken any medication to alleviate the pruritus.

**Figure 2:**
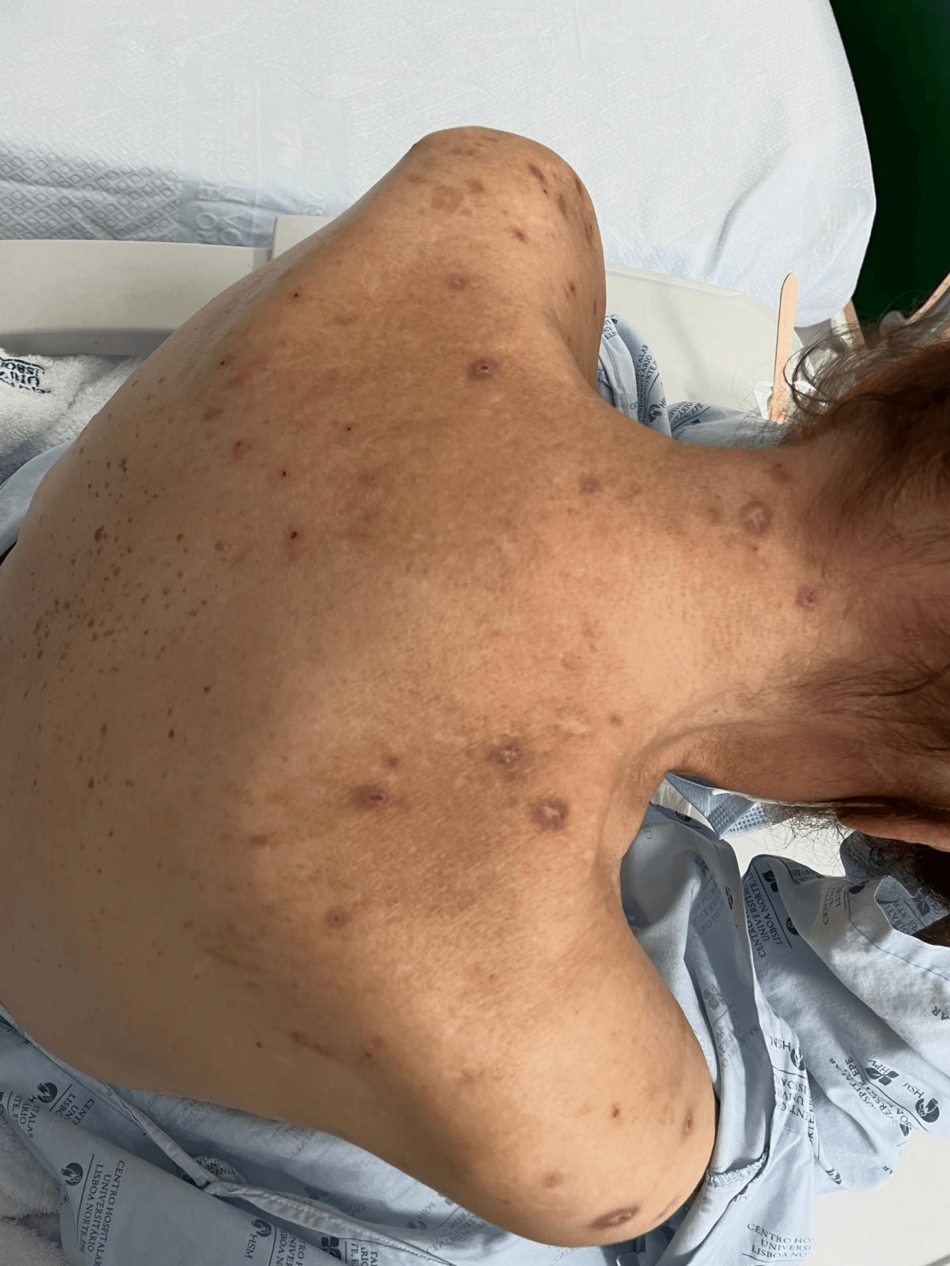
Lesions of prurigo nodularis in a patient with Waldenström macroglobulinemia.

On physical examination, the patient appeared pale and exhibited papular erythematous/brownish lesions predominantly affecting the upper extremities, as well as the back (Figure 2) and lower extremities.

Laboratory tests revealed severe anemia (hemoglobin = 5 g/dL), iron deficiency, leukocytosis with dysmorphic leukocytes, elevated CRP, elevated immunoglobulin M (IgM >5,000 mg/dL), and an increased kappa/lambda (K/L) ratio (Table 1).

**Table 1:** Laboratory evaluation on admission.

Laboratory evaluation	Value	Normal range
Hemoglobin	5.0 g/dL	12.0–15.3 g/dL
Leukocytes	27,300/µL	4,000–11,000/µL
Iron	32 µg/dL	33–193 µg/dL
Transferrin saturation	13%	20–40%
Ferritin	95 ng/mL	13–150 ng/mL
C-reactive protein	6.28 mg/dL	<0.5 mg/dL
Immunoglobulin M	5,145 mg/dL	40–230 mg/dL
Immunoglobulin G	697 mg/dL	700–1,600 mg/dL
Immunoglobulin A	51 mg/dL	70–400 mg/dL
K	945 mg/dL	155–401 mg/dL
L	64 mg/dL	93–242 mg/dL
K/L	14.77	1.29–2.61

**Figure 3:**
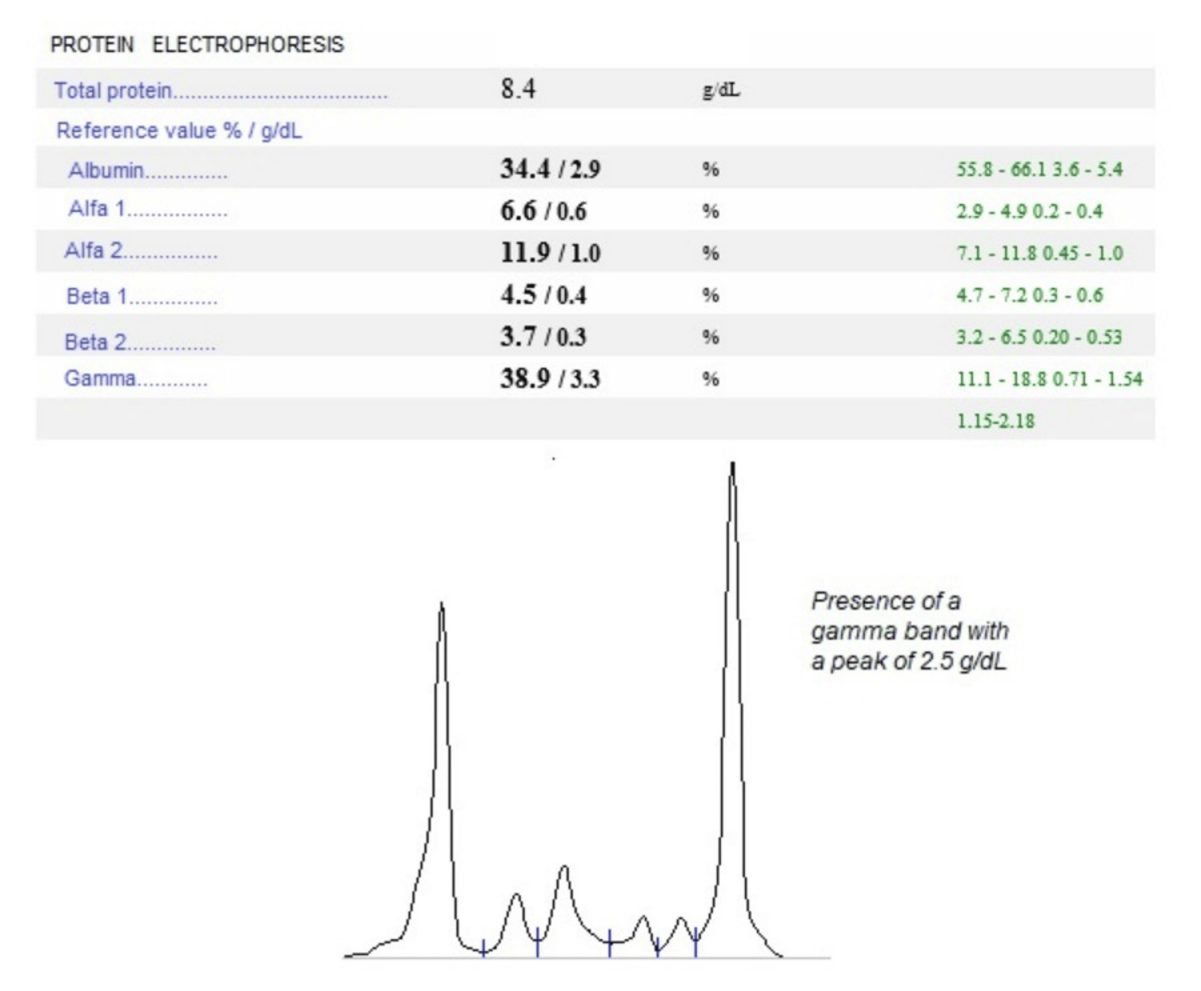
Protein electrophoresis findings.

Protein electrophoresis revealed a gamma fraction peak of 2.5 g/dL, consistent with a monoclonal band (Figure 3).

A CT scan showed no evidence of lymphadenopathy or organomegaly. A skin biopsy also showed lichenification and ulceration, suggesting the diagnosis of PN. A bone marrow biopsy confirmed small B-cell non-Hodgkin lymphoma, with an immunohistochemical profile consistent with lymphoplasmacytic lymphoma.

Based on these findings, a diagnosis of Waldenström macroglobulinemia was established, with significant bone marrow infiltration identified as the underlying cause of severe anemia.

Despite reporting headaches, the patient exhibited no other signs or symptoms of hyperviscosity syndrome, such as visual disturbances, dizziness, nausea, vomiting, dyspnea, chest pain, cognitive impairment, or pulmonary infiltrates on chest X-ray. She also denied sensory symptoms indicative of polyneuropathy, such as numbness, burning pain, or cold intolerance in the extremities.

Pruritus was controlled with hydroxyzine and high-potency topical corticosteroids until chemotherapy was started. Therapy was initiated with cyclophosphamide and dexamethasone. After the first cycle of chemotherapy, there was a complete resolution of pruritus, and the appearance of new skin lesions ceased. However, nodular cicatricial skin lesions persisted for approximately six months in the previously affected areas.

PN lesions are not always fully reversible. While treatment can significantly reduce the appearance and symptoms, including itching, complete resolution of the lesions may not always occur, especially in chronic or severe cases. The ability of the lesions to reverse depends on factors such as the underlying cause, how long the condition has been present, and the effectiveness of the treatment [7].

## Discussion

PN is often considered a primary dermatologic condition; however, its etiology can be associated with underlying systemic, neuropathic, or psychiatric factors (Table 2) that must be systematically excluded [7-9]. Medications have also been reported as a cause of PN. There have been reports implicating mainly cancer therapy agents in cutaneous side effects. In a study by Biswal et al., 0.8% (n = 3) of patients treated for cancer developed PN. Specifically, pembrolizumab, paclitaxel, and carboplatin have been associated with the development of PN. With these therapy agents, it is thought that the persistent activation of the immune system contributes to the pathogenesis of PN [8].

**Table 2:** Conditions associated with prurigo nodularis.

Category	Associated conditions
Dermatologic	Atopic dermatitis, psoriasis, urticaria, lichen planus, dermatitis herpetiformis, xerosis cutis, keratoacanthomas, bullous pemphigoid
Hematologic	Hodgkin lymphoma, non-Hodgkin lymphoma, multiple myeloma, leukemia
Oncologic	Skin malignancy (squamous cell carcinoma, basal cell carcinoma, melanoma), lung cancer, breast cancer, head and neck malignancies
Autoimmune	Celiac disease, inflammatory bowel disease, type 1 diabetes mellitus, thyroid disease
Infectious	HIV, hepatitis C, *Mycobacterium tuberculosis* and *mucogenicum*, *Ascaris lumbricoides*, *Helicobacter pylori*, *Strongyloides stercoralis*, and herpes zoster
Neuropathic	Small-fiber neuropathy, multiple sclerosis, post-herpetic neuralgia
Psychiatric	Obsessive-compulsive disorder, delusional parasitosis
Other diseases	Chronic renal failure, heart failure, cerebrovascular disease, coronary heart disease, chronic obstructive pulmonary disease, iron deficiency anemia, primary biliary cholangitis
Iatrogenic	Pembrolizumab, paclitaxel, carboplatin, ACE inhibitors, statins

In both of the presented cases, PN was diagnosed after a diagnostic workup excluded other potential causes. In Case 1, considering a young male, infectious causes were excluded as well as autoimmune disorders such as primary biliary cholangitis. As the inguinal adenopathy was detected early, the diagnostic approach prioritized the evaluation of lymphomas. In Case 2, the initial laboratory assessment, including a complete blood count, promptly identified severe anemia. Further evaluation through protein electrophoresis revealed the presence of a monoclonal peak. Additionally, common chronic diseases at this age, including renal and cardiac disease, as well as other neoplasms, were excluded. Ultimately, the underlying hematologic malignancies of Hodgkin lymphoma and Waldenström macroglobulinemia were, respectively, revealed.

To identify systemic causes of PN, we propose a structured diagnostic approach, especially for patients without a history of underlying dermatoses. Evaluation should include complete blood cell count with differential, complete metabolic panel, hemoglobin A1c, kidney function tests, liver function tests, thyroid function tests, and iron studies, as well as screening for HIV and hepatitis C virus. Eventually, serum/urine protein electrophoresis, stool studies for ova and parasites, and imaging studies (CT, MRI, radiographic) can be performed as indicated by history or physical examination. Although PN is a clinical diagnosis, biopsies are often warranted for lesions that do not respond to first-line therapies or those with secondary complications such as bleeding or ulceration [9].

PN lesions are typically symmetrically distributed in highly scratchable areas, such as the extensor surfaces of the limbs and trunk, while being less frequent in harder-to-reach areas, such as the mid-upper back, where the “butterfly sign” can be observed [10,11].

In these cases, the lesions followed this classical pattern, predominantly affecting the extremities and back. This characteristic distribution further supports the clinical diagnosis of PN and differentiates it from other dermatologic conditions with more generalized or atypical presentations. Additionally, the histopathological findings of the biopsy supported the diagnosis of PN.

Hodgkin lymphoma, particularly the nodular sclerosis subtype, is known to trigger various cutaneous manifestations, often in the form of generalized pruritus or nodular lesions. The pathophysiology behind this connection is likely multifactorial. First, the systemic inflammatory cytokines produced by lymphoma cells, such as IL-4, IL-5, and TNF-α, can increase pruritus and facilitate the development of pruritic nodules. Additionally, the lymphatic involvement in Hodgkin lymphoma may lead to the accumulation of these inflammatory mediators in the skin, thereby exacerbating the cutaneous symptoms [12].

Similarly, in the second case of Waldenström macroglobulinemia, the elevated serum IgM levels likely contributed to the development of PN through a combination of direct deposition of immunoglobulins in the skin (cutaneous macroglobulinosis) and the systemic inflammatory effects of the disease. IgM paraproteins in WM can lead to hyperviscosity syndrome, a condition that affects multiple organ systems, including the skin. Cutaneous manifestations of hyperviscosity syndrome include livedo reticularis and the development of nodules or ulcers, which can sometimes be confused with PN. Although the relationship between Waldenström macroglobulinemia and PN is less well studied than in Hodgkin lymphoma, the underlying inflammatory milieu in Waldenström macroglobulinemia, combined with immune dysregulation, could also contribute to pruritus and nodular lesion formation. Furthermore, increased IgM levels have been associated with pruritic skin conditions, making it an important factor to consider when evaluating patients with cutaneous symptoms and systemic signs of Waldenström macroglobulinemia [13-15].

The association between chronic prurigo and hematologic malignancies, particularly Hodgkin lymphoma, has been documented in the literature. A previously reported case closely resembles the present one, as the initial diagnosis was also missed due to suspicion of scabies, with the lesions resolving shortly after chemotherapy initiation [13]. Another study from Dermatology Research and Practice further reinforces the relevance of PN as a rare cutaneous manifestation of HL, underscoring the importance of clinician awareness for early recognition and timely intervention [14]. Additionally, we identified a case of cutaneous macroglobulinosis occurring in the context of Waldenström macroglobulinemia, with clinical similarities to PN [15]. However, no previous reports directly linking Waldenström macroglobulinemia to PN were found in the literature. In Case 2, the skin biopsy supported the diagnosis of PN, and the presence of Waldenström macroglobulinemia was established. Thus, to our knowledge, this represents the first documented case of Waldenström macroglobulinemia with confirmed prurigo nodularis, further emphasizing the need for heightened clinical suspicion in similar presentations.

In the cases presented, the initiation of chemotherapy led to significant improvements in the pruritus and resolution of the cutaneous nodules. This aligns with the findings of other reports, where patients with lymphoma-related PN experienced resolution of skin lesions following chemotherapy or radiation therapy [16].

Ongoing research into the immunological and inflammatory pathways involved in PN could help clarify the exact mechanisms by which these malignancies contribute to its development. Further studies are needed to determine the optimal management strategies for patients with PN associated with malignancy, including the potential role of novel immunomodulatory therapies that target the underlying inflammatory pathways.

The internist plays a key role in the evaluation and management of PN, given its potential association with systemic diseases such as chronic kidney disease, diabetes, and malignancies. Beyond identifying underlying conditions, the internist helps rule out differential diagnoses, manage comorbidities such as anxiety and depression, and oversee treatment selection, monitoring, and adjustments. Their involvement ensures a comprehensive approach, improving diagnostic accuracy, optimizing therapy, and preventing complications.

## Conclusions

PN should be considered a potential cutaneous manifestation of systemic malignancy, especially in cases with severe, persistent, or treatment-refractory pruritus. Clinicians should consider evaluating for underlying hematologic malignancies, such as Hodgkin lymphoma and Waldenström macroglobulinemia, when PN is accompanied by systemic symptoms such as weight loss, fatigue, and lymphadenopathy. Early diagnosis and treatment of the underlying malignancy are crucial for improving patient outcomes, as systemic treatments targeting the malignancy can lead to the resolution of both the malignancy and the associated pruritic skin lesions.
